# Driving and dementia: a clinical decision pathway

**DOI:** 10.1002/gps.4132

**Published:** 2014-05-27

**Authors:** Kirsty Carter, Sophie Monaghan, John O'Brien, Andrew Teodorczuk, Urs Mosimann, John-Paul Taylor

**Affiliations:** 1 Institute for Ageing and Health, Newcastle UniversityNewcastle upon Tyne, UK; 2 London and Maudsley NHS Foundation TrustLondon, UK; 3 Department of Psychiatry, University of CambridgeCambridge, UK; 4 Department of Old Age Psychiatry, University Hospital of PsychiatryBern, Switzerland

**Keywords:** driving, dementia, pathway, ageing

## Abstract

**Objective:**

This study aimed to develop a pathway to bring together current UK legislation, good clinical practice and appropriate management strategies that could be applied across a range of healthcare settings.

**Methods:**

The pathway was constructed by a multidisciplinary clinical team based in a busy Memory Assessment Service. A process of successive iteration was used to develop the pathway, with input and refinement provided via survey and small group meetings with individuals from a wide range of regional clinical networks and diverse clinical backgrounds as well as discussion with mobility centres and Forum of Mobility Centres, UK.

**Results:**

We present a succinct clinical pathway for patients with dementia, which provides a decision-making framework for how health professionals across a range of disciplines deal with patients with dementia who drive.

**Conclusions:**

By integrating the latest guidance from diverse roles within older people's health services and key experts in the field, the resulting pathway reflects up-to-date policy and encompasses differing perspectives and good practice. It is potentially a generalisable pathway that can be easily adaptable for use internationally, by replacing UK legislation for local regulations. A limitation of this pathway is that it does not address the concern of mild cognitive impairment and how this condition relates to driving safety. © 2014 The Authors. *International Journal of Geriatric Psychiatry* published by John Wiley & Sons, Ltd.

## Introduction

Driving is increasingly an integral part of human life, particularly in developed countries, providing autonomy and other psychosocial benefits (Hiscock *et al*., 2002). Concurrent with economic development, the number of drivers is increasing worldwide, and this changing demographic is also mirrored by an increasingly aged population who drive, particularly the number of female older drivers. In the UK, at present, 78% of people older than 60 years and 54% of people older than 70 years hold a current driving licence (National Traffic Survey, 2009). Driving allows older people greater freedom to access different aspects of society. This is particularly important if they are limited in their physical mobility or are socially isolated, for example, in a rural community, where public transport links may be sporadic (O'Neill, 2010). However, with age comes an increased risk of dementia, and studies have demonstrated that those with a diagnosis of dementia are at an increased risk when driving (e.g. Man-Song-Hing *et al*., 2007). They are more likely to become lost (Eby *et al*., 2012), travel too slowly (Eby *et al*., 2012), not wear a seat belt (Eby *et al*., 2012) and be involved in a collision (Breen *et al*., 2007). In addition, they can present an elevated accident risk (Breen *et al*., 2007; Marshall, 2008). Driving risk increases, depending on disease severity (Iverson *et al*., 2010).

In the UK, currently, 1 in 14 people older than 65 years and 1 in 6 people older than 80 years have a diagnosis of dementia, and this is set to rise by 2025, to over one million people in the UK (Dementia UK, 2007). This demographic expansion in people with dementia is worldwide, and it is likely that 115 million people will be living with dementia by 2050 (World Alzheimer Report, 2009). Therefore, increasingly, the numbers of those with dementia who drive represent a major and increasing problem. Specific challenges are raised, which include the underdiagnosis of dementia and consequently the lack of awareness of many people who drive and their families without knowing they have dementia. However, early diagnosis raises the challenging question of whether a patient is fit to drive, and there are several key areas to consider.

### Is a patient with dementia safe to drive?

For the clinician, the task of determining whether a patient with dementia has the ability to continue to drive safely may be problematic. The on-road assessment at an accredited mobility centre for drivers with cognitive impairment, in the UK, is recognised by the British Psychological Society (2001) as being the ‘gold standard’ (Box 1), and the importance of the on-road assessment is further supported by old age psychiatrists (Naidu and Mckeith, 2006) as being the most popular suggestion as to how to address driving ability. Areas of clarity do exist; for example, patients with moderate to severe dementia are not fit to drive, and many patients with dementia surrender their licence voluntarily. However, there is no clear definition of early or mild dementia, although Iverson *et al*. (2010) do make a suggestion to deal with this, for example, using the Clinical Dementia Rating Scale, caregivers' rating of driving ability, a history of crashes, reduced mileage and a mini-mental state examination of <24 to identify patients who are at increased risk of unsafe driving. Also, fitness to drive should be based not only on dementia severity alone but also on other relevant factors such as vision, hearing, head turning ability and daytime sleepiness (Mosimann *et al*., 2012), which either may associate with dementia or be independent from it or indeed synergistically act with the cognitive impairment to affect driving ability. Many studies have investigated the role that neuropsychological testing has in evaluating an individual's safety to drive. It has been variously reported that a general cognitive test battery (e.g. Dawson *et al*., 2009; Lincoln *et al*., 2010), selective attention tasks (e.g. Ducheck *et al*., 1997), maze test performance (e.g. Ott *et al*., 2003) and visuospatial tasks (e.g. Silva *et al*., 2009) can be utilised to either predict safe driving behaviour or be used to supplement the clinician's judgement. However, numerous studies refute the efficacy of cognitive testing as a measure of driving ability (e.g. Bieliauskas *et al*., 1998; Brown and Ott, 2004; Molnar *et al*., 2006), and as no consensus has been reached in this area, it is not possible to offer definitive guidance to the clinician on what neuropsychological tests are best in this regard. Nevertheless, given that an assessment of cognition and activities of daily living are required when diagnosing dementia, this can at least help to detect those with moderate to severe disease (Wagner *et al*., 2011) in whom the decision to advise driving discontinuation is much more straightforward.


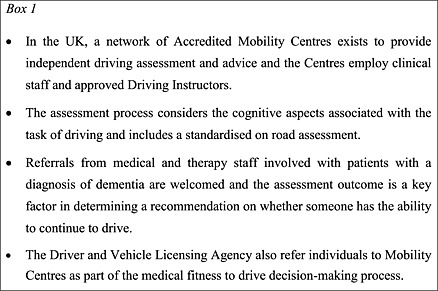


The use of driving simulators, although an under-researched area, may become a promising tool for assessing fitness to drive in the future. A retrospective study by Lee *et al*. (2003a, 2003b) indicated that individuals at increased risk of a crash could be identified using a PC-based driving simulator, while a comparison of a simulator with an on-road test (Lee *et al*., 2003a, 2003b) has supported the validity of the driving simulator. However, the availability of driving simulators is not widespread, there are a large number of simulator packages and no common standard, and assessment using these requires additional expertise. In addition, there can also be problems with simulator sickness (Classen *et al*., 2011), which can present a challenge, particularly in older drivers (Brooks *et al*., 2010).

### Differing legislative requirements

In Europe, no specific citation is made of dementia in the European directive (91/439/EEC) regarding standards of mental and physical capability to drive leading to differing interpretations between member states (Breen *et al*., 2007). In the UK, patients are required to inform the Driver and Vehicle Licensing Agency (DVLA, 2011) when a diagnosis of dementia is received, and physicians are recommended to report to the DVLA those likely to continue driving despite being advised not to when it is no longer safe. In contrast, in the Netherlands, medical fitness to drive is assessed at licence renewal or based on self-report from the individual. If a dementia is reported to the licensing authority, depending on disease severity and progression, patients can undergo examination from a neurologist/neuropsychologist and have an expert driving assessment (CBR, 2000), and as an outcome from this, they may be deemed temporarily suitable to retain their licence, for usually not more than 5 years.

Outwith the EU, legislation is equally varied on how of a diagnosis of dementia is reported to the appropriate licensing authority. In Canada, for example, regulations are state specific, and most states require mandatory reporting of medically unfit drivers, although dementia is not specifically mentioned (CMA drivers guide, 2006). The US legislation also differs from state to state but most do not require mandatory reporting by clinicians (exceptions include, for example, California, Pennsylvania, Delaware, Oregon, Indiana, Arizona and New Mexico; with only California and Pennsylvania specifically mentioning Alzheimer's disease) (Rapoport *et al*., 2007). Australia requires the patient to inform the licensing authority of any permanent long-term injury or illness that affects safe driving ability, but again no specific mention of dementia is made (Angley, 2001; Austroads, 2003). New Zealand only requires reporting if a patient is likely to continue driving after they have been advised to cease (NZTA, 2009). However, all worldwide legislation has a propensity to recommend that a diagnosis of dementia alone is not adequate enough to withdraw an individual's licence to drive, but it offers no guidance on what constitutes fitness to drive, for those expected to make this recommendation.

### Lack of guidance in how to deal with driving cessation in dementia

Many patients who receive a diagnosis of dementia continue to drive (Adler *et al*., 2005), with numbers estimated at over 40% (Adler and Kuskowski, 2003), and it has been reasoned that tackling the issue of driving and driving cessation should be a collaborative process between the healthcare professional physician, patients and their families (O'Neill, 2010). However, family dynamics can make driving cessation problematical. Relatives who rely on the patient for transport in routine activities such as shopping, recreation and childcare are more likely to continue their dependence on the patient driving, despite impairment (Adler *et al*., 2000a, 2000b), and so be less willing to work in partnership with the physician (Adler *et al*., 2000a, 2000b). From a patient's perspective, the cessation of driving can also lead to a loss of independence (Adler *et al*., 2000a, 2000b), increased dependence on family members (Taylor and Tripodes, 2001) and/or a change in living circumstances (Adler *et al*., 2000a, 2000b). The loss of driving ability can lead to decreased life satisfaction (Cutler, 1975) and increased isolation (Marottoli *et al*., 2000) and depression (Ragland *et al*., 2005). Crucially, patients and their families may often find that there is little available in the way of viable alternatives (e.g. Taylor and Tripodes, 2001; Arai *et al*., 2011).

Generally, given the onus is typically on clinical staff, usually the physician, to make a judgement on the patient's competence to drive (Brown and Ott, 2004), this can lead to a conflict between the patient and the clinician with associated ramifications and effects on the therapeutic alliance between patient and clinician. A complicating factor is the need to not only make a judgement at the initial stage of the therapeutic relationship but also take a long-term outlook because of the degenerative nature of dementia (O'Neill, 2010).

The lack of guidance and ambiguity in how to deal with the issue of driving and the patient with dementia may lead to a clinician's reluctance to tackle the issue. Although key guidelines have been issued for dementia in the UK and Europe (e.g. NICE, 2006; Hort *et al*. 2010 ), these have failed to satisfactorily address the issue of driving, with these guidelines only briefly mentioning that medicolegal issues including driving need to be addressed but offering no supporting guidance for the clinician.

In summary, there is a great deal of uncertainty on how patients with a dementia diagnosis, who wish to continue to drive, should be managed. Hunter *et al*. (2009) support the need for an objective way of dealing with the issue and advises that a ‘co-operative approach between the clinical team responsible for the person's on-going care and a driving assessment team is the best way of dealing with difficult issues in this range of diseases’.

No real consensus exists on how cases should be handled at either local or national level, and many NHS trusts are now recognising the urgent need to develop driving and dementia protocols. However, although there exists some literature addressing fitness to drive in dementia (e.g. Mosimann *et al*., 2012; Iverson *et al*., 2010) and a “toolkit” guide developed in Canada (Dementia network of Ottawa, 1997), there is no generally accepted care pathway to guide clinicians and people with dementia.

**Figure 1:**
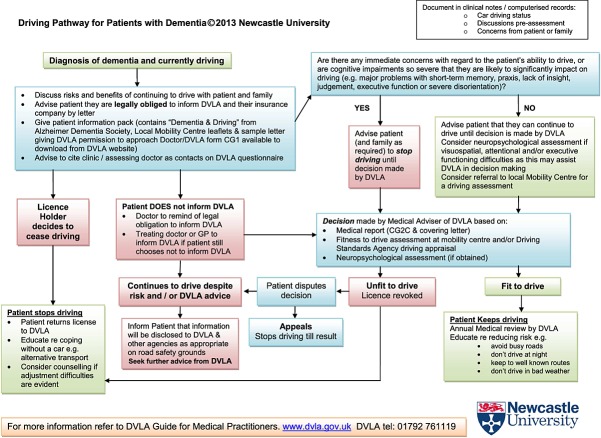
Driving pathway for patients with dementia (available to download from http://research.ncl.ac.uk/driving_and_dementia).

Therefore, our aim was to provide a pathway that offered clarity in managing patients with dementia who drive, with a secondary aim of improving road safety and enabling those who are safe to drive to continue to do so. We report the development of such a pathway (Figure 1) in the UK—the purpose of which was to bring together current UK legislation, good clinical practice and appropriate management aspects into a simple pathway that could be rapidly and easily applied across a range of healthcare settings and also be utilised by individuals with limited experience of managing this issue.

## Method

The pathway was initially constructed within an experienced multidisciplinary clinical team in a busy Memory Assessment Service (comprising a professor of old age psychiatry, two senior clinical academics, an experienced clinical psychologist and an assistant psychologist). A process of successive iteration, and consensual discussion within the group, developed a draft pathway. Once the initial pathway had been drafted, a survey (respondents were asked to provide ratings for the utility of the pathway, how likely they would be to use it in practice and how much it clarified the issue for them, as well as provide suggestions for alterations or inclusion) of individuals, with 29 respondents, from a wide range of regional networks (the North East Regional Old Age Psychiatry Network, the North East branch of Psychology Specialists working with Older People, North East branch of Psychology Specialists working with Older People—Neuro-Special Interest Group and the Newcastle and South Tyneside Older Adult Community Mental Health Team) and diverse clinical backgrounds (medicine and psychiatry (15), nursing ( 4), psychology (8) and occupational therapy (2)) helped refine the pathway. Participants were asked to provide ratings for the utility of the pathway, how likely they would be to use it in practice and how much it clarified the issue for them, as well as provide suggestions for alterations or specific inclusions. Finally, the pathway was further shaped following discussion with key external stakeholders, that is, North East Drive Mobility (the Accredited Mobility Centre in the North East of England), the UK Forum of Mobility Centres and the Driver and Vehicle Licensing Agency, into its final format.

## Results

### The pathway

The completed pathway (Figure 1) consists of a logically ordered flow diagram, which is colour coded for ease of use. It provides a step-by-step process to guide the user through possible pathways an individual clinician may take, beginning with their diagnosis and initial discussion relating to driving. The user is then guided through steps to take if there are concerns regarding the individual's ability to drive. If the patient decides to cease driving, the user is guided to the appropriate legal and supportive actions to take. If the patient decides to continue driving, the user is directed to an appropriate course of action via the pathway, which is communicated to the person and, if appropriate, their family. The successive stages then channel the user through the relevant clinical and legal procedures, in a logical progression, pending the outcome of the DVLA decision-making process.

It should be recognised, however, that this pathway is not presented as a stand-alone item, but it is the central aspect of an overall support package that was developed by the aforementioned team. The full ‘driving pack’ contents are presented and described in Table 1 (full pack available to download from http://research.ncl.ac.uk/driving_and_dementia).

**Table 1:** Contents of driving pack

Content	Description
Introduction to pack	A leaflet informing the user on the contents of the pack and guidance on use
The pathway	A copy of the pathway itself
Patient information leaflet	Gives guidance for the patient and answers common questions around the process and offers support and guidance for alternative methods of transport
DVLA guidance	Official guidelines from the DVLA on the process of reporting a diagnosis
Local mobility leaflet	Gives information from the local drive mobility centre on what services can be offered
Template letter	A template letter for use by clinicians and patients to inform the DVLA of a diagnosis
Discussion guidance	A template sheet offering guidance questions that can be asked of the patient and family, to guide discussion around the issue
DVLA CG1 form	DVLA medical information form for patients to complete, giving details of medical condition
DVLA surrender of licence form	Form for patients to complete and return to the DVLA if they decide to surrender their licence

## Discussion

We present a clinical pathway for patients with dementia, which was developed following a robust process with input from key experts in the field. The overall aim was to address the uncertainty that exists on how patients with a dementia diagnosis, who wish to continue to drive, should be managed. By this process, we have drawn together current UK legislation, good clinical practice and appropriate management aspects into a simple care pathway that could be rapidly and easily applied across a range of healthcare settings and also be utilised by individuals with limited experience of managing this issue.

A range of clinical pathways are available through the National Institute for Health and Care Excellence and bring together clinical guidelines, interventional procedures, public health guidance and quality standards into a logical flow diagram for users (NICE, 2011). Pathways are accessible for a range of issues, from blood disorders to mental health. A dementia pathway has been established (NICE, 2011), and although this is a generalised pathway, the driving and dementia pathway would complement this, for example, by fitting into, promoting independence and maintaining function section.

A strength of this pathway is the multidisciplinary approach utilised in its development. The developers were able to integrate the latest guidance from diverse roles within older people's service and key experts in the field, resulting in a pathway that reflects up-to-date policy and encompasses differing perspectives and good practice. This procedure enhances the efficacy of the pathway as a general tool that can be utilised across all disciplines within service.

The pathway also provides the framework for a uniform approach across services, as the pathway is not a stand-alone tool. It forms part of a driving pack, that contains further information and scaffolds the pathway, for example, information on local mobility centres; and alternative forms of transport, and is supported by relevant patient information on pre and post driving cessation advice. It is hoped that this can facilitate discussion with patients and families and enhance a collaborative approach (O'Neill, 2010), between clinician, patient and family when discussing the process of continuing to and eventual cessation of driving. The use of the pathway as part of a driving pack will also serve to normalise the process of dealing with driving in the clinical setting, as an on-going aspect of management of the disease (Adler *et al*., 2000a, 2000b), and aid in addressing the issue of viable alternatives (Taylor and Tripodes, 2001; Arai *et al*., 2011).

This pathway was developed in the UK, taking into account UK practice and legislation; however, it is potentially a generalised pathway that can be easily adaptable for use worldwide, by substituting UK-based (DVLA) legislation for local regulations. The multidisciplinary method utilised in development enables it to be integrated into differing service approaches, internationally. This may also facilitate in addressing the lack of guidance and clarification that emerged, when investigating European, American, Australian and New Zealand policy.

Particular limitations emerged during the development of this pathway. The first is that in the area of driving and dementia, there is a lack of coherency in the field in terms of theory and empirical evidence, and thus, our pathway is informed on the basis of expert clinical judgement and consensus opinion. And although we recognise this is not as strong as empirical evidence, it was ratified by a range of respondents as clinically useful. However, where possible, the advice given is based upon previous research (e.g. Chu (1994) found that older drivers do report that driving at night and at peak hours is more problematical for them. Also, reduced crash rates for older drivers at evenings and weekends (Stutts and Martell, 1992) suggest that older drivers avoid driving at these times, so it would be reasonable to suppose that advising a reduction in these behaviours would reduce risk). Therefore, an important next step in implementing the pathway into practice would be to carry out formal service evaluations to test the utility of the pathway and compare it with usual practice. Also, in our consultation process, a number of participants expressed the desire for a definitive neuropsychological domain that can be tested, or a cognitive test battery that can be utilised, to determine an individual's ability to drive. However, the evidence base for this is lacking, and the use of neuropsychological testing for this purpose remains a controversial area, with no consensus reached on what areas or tests are particularly useful. Which neuropsychological domains correlate with drive ability is an area for further research, and the development of a short test battery, which can be used in clinic, would be apposite and useful in addressing immediate concerns. In particular, with the increasing availability of technology, the utility of driving simulation shows promise as a relevant tool and would be a useful area for exploration although access and cost may be major barriers. Our pathway does not seek to determine the driving ability of patients but to offer best practice guidance to clinicians and clarification on the issues surrounding driving with dementia.

A further potential limitation of this pathway is that it does not address the concern of mild cognitive impairment (MCI) (Budson and Solomon, 2012; Petersen, 2004) and how this condition relates to driving safety. The diagnosis of MCI remains a contentious area, because the label is not necessarily indicative of an underlying neurodegenerative process. Furthermore, it seeks to medicalise a mild impairment, which is defined as having little or no functional impact. Importantly, people in receipt of this diagnosis may not experience any further decline or necessarily progress to a dementia (Whitehouse and Moody, 2006), and some may revert to normal cognitive function on reassessment (Koepsell and Monsell, 2012). Thus, it may be inappropriate to label such individuals medically and potentially from a legislative perspective, as impaired, with regard to driving. Current guidance, practice and legislation are not sufficient in providing a clarified and unified approach managing this controversial question. Further work is needed in this area, with next steps being the production of a pathway to provide clarity and best practice surrounding those with MCI.

## Conflict of interest

None declared.

**Key points**

- Individuals with a diagnosis of dementia are at increased risk when driving.
- Currently, no consensus guidance exists.
- The pathway is designed to address this disparity.

## Ethics statement

This paper reports a clinical service development and clinical opinion document. In this context, it is not a formal research study and therefore did not require specific institutional or ethical approval for its completion.
